# Identification of Lipid Biomarkers for Chronic Joint Pain Associated with Different Joint Diseases

**DOI:** 10.3390/biom13020342

**Published:** 2023-02-09

**Authors:** Spiro Khoury, Jenny Colas, Véronique Breuil, Eva Kosek, Aisha S. Ahmed, Camilla I. Svensson, Fabien Marchand, Emmanuel Deval, Thierry Ferreira

**Affiliations:** 1 Université de Poitiers, Laboratoire Lipotoxicity and Channelopathies (LiTch)—ConicMeds, 86073 Poitiers, France; 2 Université de Poitiers, Laboratoire PRéTI, 86073 Poitiers, France; 3 Université Côte d’Azur (UCA), UMR E-4320 MATOs CEA/iBEB/SBTN, Faculté de Médecine, CEDEX 2, 06107 Nice, France; 4 Service de Rhumatologie, Hôpital Pasteur, CHU de Nice, 06000 Nice, France; 5 Department of Clinical Neuroscience, Karolinska Institutet, 17165 Solna, Sweden; 6 Department of Surgical Sciences, Uppsala University, 75185 Uppsala, Sweden; 7 Department of Molecular Medicine and Surgery, Karolinska Institutet, 17176 Stockholm, Sweden; 8 Department of Physiology and Pharmacology, Center for Molecular Medicine, Karolinska Institutet, 17165 Solna, Sweden; 9 Université Clermont Auvergne, Inserm U1107 Neuro-Dol, Pharmacologie Fondamentale et Clinique de la Douleur, 63001 Clermont-Ferrand, France; 10 Université Côte d’Azur, CNRS, IPMC, LabEx ICST, FHU InovPain, 06560 Valbonne, France

**Keywords:** lipidomics, chronic joint pain, synovial fluid, inflammation, metabolic status

## Abstract

Lipids, especially lysophosphatidylcholine LPC16:0, have been shown to be involved in chronic joint pain through the activation of acid-sensing ion channels (ASIC3). The aim of the present study was to investigate the lipid contents of the synovial fluids from controls and patients suffering from chronic joint pain in order to identify characteristic lipid signatures associated with specific joint diseases. For this purpose, lipids were extracted from the synovial fluids and analyzed by mass spectrometry. Lipidomic analyses identified certain choline-containing lipid classes and molecular species as biomarkers of chronic joint pain, regardless of the pathology, with significantly higher levels detected in the patient samples. Moreover, correlations were observed between certain lipid levels and the type of joint pathologies. Interestingly, LPC16:0 levels appeared to correlate with the metabolic status of patients while other choline-containing lipids were more specifically associated with the inflammatory state. Overall, these data point at selective lipid species in synovial fluid as being strong predictors of specific joint pathologies which could help in the selection of the most adapted treatment.

## 1. Introduction

Chronic joint pain is a major health problem, affecting a large number of people worldwide. It has been estimated that in 2050, 130 million people will suffer from osteoarthritis (OA) alone, of whom 40 million will be severely disabled (A Public Health Approach to Innovation Update on 2004 background paper, 28 January 2013. World Health Organization. Available from: https://www.who.int/medicines/areas/priority_medicines/BP6_12Osteo.pdf accessed on 5 November 2021) [1]. The activation of acid-sensing ion channel 3 (ASIC3), a sub-type of ASICs expressed within the lipid bilayer of the cell membrane of mainly peripheral neurons, has been demonstrated to play a key role in this type of pain [2,3]. ASIC3 is usually activated by extracellular protons under acidic conditions [4]. However, several other factors, including certain endogenous lipid species, such as the choline-containing species lysophosphatidylcholine (LPC) and a specific free fatty acid, namely arachidonic acid (AA), can either activate and/or potentiate ASIC3 under non-acidic physiological conditions [2,5]. Both LPC and AA can be concomitantly released after hydrolysis of the membrane phospholipid (PL) phosphatidylcholine (PC) by phospholipase A2 [6].

Chronic joint pain is observed in several musculoskeletal diseases either from non-inflammatory or inflammatory origins, e.g., cartilage degeneration, crystal deposition, infection, rheumatoid arthritis, psoriatic rheumatism, and spondyloarthritis. Systemic inflammation is well known to correlate with high concentrations of circulating C-reactive proteins (CRP) [7]. Interestingly, CRP is known to bind to choline-containing lipids both in human and mouse, and subsequently activate the complement system, ultimately promoting inflammation [8,9,10]. Therefore, choline-containing lipid species are likely to act as central players in chronic joint pain, both as amplifiers of the inflammatory process and as direct activators of ASIC3 channels.

Synovial fluid (SF) is a complex mixture considered as an ultrafiltrate of blood plasma, and contains molecules that provide low-friction and low-wear properties to articulating cartilage surfaces. It is primarily composed of hyaluronan, lubricin, proteinase and collagenases [11]. SF also contains lipids, but the concentrations of these compounds are still generally less than those observed in the plasma of normal SF. However, alterations in the composition of SF and changes in the level of its constituents, including lipids, can be an indicator of different pathologies. The lipid composition of synovial fluids from post mortem control subjects with no history of joint disease and patients suffering from chronic joint pain has been the object of several studies [12,13,14,15]. However, if significant modifications in the amounts of selective lipid species have been reported (see below), no clear correlations have been established yet between observed variations and specific clinical parameters. Moreover, none of these studies proposed a large lipid profiling in different joint diseases, since most of them focused either on OA or rheumatoid arthritis (RA).

Therefore, our aim was first to identify and quantify potential lipid biomarkers, with a selective emphasis on choline-containing lipid species, in the synovial fluid of patients suffering from different painful joint disorders, as compared to controls. Lipidomic analyses, based on high-resolution mass spectrometry (HR-MS) experiments, revealed selective lipid changes in patients’ synovial fluids according to the type of the joint pathology. Correlational analyses were also performed between lipid levels in synovial fluids and several patient clinical characteristics, including gender, body mass index (BMI), age, metabolic status, and systemic inflammatory state. These analyses mainly highlighted correlations between the levels of specific lipids in the synovial fluids of patients and their metabolic and inflammatory status.

## 2. Material and Methods

### 2.1. Synovial Fluid Samples

#### 2.1.1. Post Mortem Controls

Ten post mortem (PM) subjects with no history of knee or hip OA or inflammatory rheumatic diseases were included as controls. Synovial fluid from the knee joint was collected during the autopsy procedure and immediately frozen at −80 °C for future analysis.

#### 2.1.2. Patients with Different Joint Diseases

Demographic data about this cohort have already been published [3]. Briefly, fifty patients (32 women and 18 men, average age, 68.6 years, range 26–94 years) suffering from different joint pathologies (rheumatoid arthritis (RA, *n* = 6), chondrocalcinosis (CCA, *n* = 12), spondyloarthritis (SPA, *n* = 5), psoriatic arthritis (PA, *n* = 4), gout (*n* = 5), and OA (*n* = 18)) were recruited from the Rheumatology Department of the Nice University Hospital in France. Age and BMI distributions in each group of patients are available in Supplementary Table S1. All subjects provided informed consent before inclusion, and the study was approved by the Nice University Institutional Review Board for Research on Human Subjects. The study has been conducted in accordance with French national regulations regarding patient consent and ethical review. The study was registered in the ClinicalTrials.gov protocol registration system (NCT 01867840). All synovial exudates were obtained from patients with acute knee joint effusion requiring joint puncture for diagnosis and/or treatment. Synovial fluid samples, which remained after biological analysis for patients’ care, were frozen at −80 °C for future lipidomic analysis.

The white blood cell count in the synovial fluids of patients (Malassez blades) was manually performed with a hemocytometric chamber by light microscopy analysis. Differential white blood cell counts were determined in synovial fluid smears by the May-Grünwald-Giemsa (MGG) staining. The MGG slide was analyzed by light microscopy under oil immersion at x50 magnification. The first 100 nucleated cells counted in random fields were subclassified as neutrophils, lymphocytes, and monocytes. The results were expressed as the number of cellular elements, leukocytes and red blood cells/mm^3^ and the percentage of polynuclear neutrophils (PNN%), lymphocytes and macrophages.

The determination of total cholesterol, HDL and triglycerides levels in the plasma of patients was performed by spectrophotometry (Cobas 8000, module 700, Roche Diagnostics, 38240 Meylan, France). The LDL level was calculated using total cholesterol and HDL levels.

### 2.2. Chemical and Lipid Standards

Chloroform (CHCl_3_), Methanol (CH_3_OH) and Formic acid (HCOOH) were purchased from Sigma Aldrich (Saint Quentin Fallavier, France). The water (H_2_O) used for lipid extraction came from Milli-Q. Lipid standards (Lyso PC 16:0, Lyso PC 13:0, Lyso PAF 16:0, PAF “from heart PC”, PC(16:0/18:1), PC 14:0 (DMPC), FA20:0 and FA17:0) were purchased from Avanti Polar Lipids via Sigma Aldrich and then prepared at the appropriate concentration and stored at −20 °C.

### 2.3. Lipid Extraction

The extraction of lipids from human synovial fluid (HSF) samples was adapted from the Folch method. Fifty microliters of control and patient exudates were diluted with water to a final volume of 1 mL. The diluted samples were then transferred into glass tubes (Pyrex Labware) and vortexed for 1 min. Lipids were extracted using 4 mL of chloroform (CHCl_3_)/methanol (CH_3_OH) (2:1, *v*/*v*) and shaking with an orbital shaker (IKA^®^ VX^®^ basic Vibrax^®^, Sigma-Aldrich) at 1500 rpm for 2 h at room temperature. After centrifugation for 10 min with a swing-out centrifuge at 410 g, the aqueous phases were eliminated and lipid-containing organic phases were supplemented with 1 mL of a 4/1 (*v*/*v*) 2N KCl/CH_3_OH solution. Samples were shaken for 10 min at 1500 rpm, centrifuged for 5 min, and the upper aqueous phases were eliminated. The resulting organic phases were complemented with 1 mL of 3/48/47 (*v*/*v*/*v*) CHCl_3_/CH_3_OH/H_2_O solution, shaken for 10 min and centrifuged for 5 min. The aqueous phases were eliminated and the organic phases containing the whole lipids were transferred into new glass tubes. Lipid extracts were then evaporated to dryness at 60 °C under a stream of nitrogen, re-dissolved in 500 µL CHCl_3_/CH_3_OH (1:2, *v*/*v*), and stored at −20 °C until further analysis.

### 2.4. Polar Lipid Purification

After lipid extraction from the synovial fluids using an adapted protocol of the Folch method [16], we evaluated whether an additional purification step of the most polar lipids from total lipid extracts could increase the extraction yield of choline-containing-lipids. For this, total lipid extracts were evaporated until dryness under a stream of nitrogen, re-suspended in 1 mL dichloromethane, and shaken for 1 min. These solutions, containing total lipids, were loaded into silica columns (BOND ELUT-SI, 100 mg 1 mL, Agilent Technologies) previously conditioned with 3 mL methanol and 2 mL dichloromethane successively. The different lipid classes were eluted from the columns using a solvent or a mixture of solvents of increasing polarity. Non-polar lipids were first eluted with 2 mL dichloromethane, and glycolipids were then eluted with 3 mL acetone. Finally, 2 mL of 50/45/5 (*v*/*v*/*v*) CHCl_3_/CH_3_OH/H_2_O solution was loaded into the columns to elute the polar lipids. The eluted fractions containing the polar lipids (especially the phospholipids) were collected into glass tubes. Solvents of these fractions were evaporated at 60 °C under a stream of nitrogen until dryness. Dry purified phospholipids were then re-suspended in CHCl_3_/CH_3_OH (1:2, *v*/*v*) and stored at −20 °C. The comparison between purified and non-purified lipid extracts led to similar levels of PC and PCp. Unexpectedly, the purification step resulted in a lower recovery of LPC species as compared to raw extracts. The analyses presented in this study were therefore performed on total lipid extracts without any additional purification step.

### 2.5. Electrospray Ionization-Mass Spectrometry Conditions for Lipids Analysis

Lipid extracts were diluted to the appropriate concentration in CHCl_3_/CH_3_OH (1:2, *v*/*v*). Optimized amounts of internal lipid standards (LPC 13:0, PC(14:0/14:0) and FA 17:0) were added to each sample in order to calculate lipid levels. Lipid extracts were analyzed by direct infusion on a SYNAPT^TM^ G2 High Definition Mass Spectrometer HDMS (Waters Corporation, Milford, MA, USA) equipped with an Electrospray Ionization Source (ESI). The flow rate for MS experiments was 5 µL × min^−1^. All full scan Mass Spectrometry experiments were acquired in profile mode over 1 min with a normal dynamic range from 300 to 1200 *m/z*. The analyzer was set to resolution mode. Analyses were carried out in positive and negative ion modes. Samples were supplemented by 1% (*v*/*v*) formic acid for positive ESI experiments. Ionization parameters for positive and negative ionization have been optimized and defined as shown in Supplementary Table S2. Under these conditions, all lipid species, including LPC, Lyso PAF, PC and plasmalogen PC (PCp) were detected as protonated ions [M+H]^+^ in the positive ion mode. The free fatty acid (AA) was analyzed in negative ion mode and was detected as [M-H]^−^. Furthermore, tandem mass spectrometry (MS/MS) experiments were conducted in positive ion mode in order to confirm the structure of lipid species. These MS/MS experiments were carried out by collision-induced-dissociation (CID), with optimized collision energy at 37 V, in a normal dynamic range.

### 2.6. Lipid Identification by MS, Calculation of Lipid Levels and Data Processing

The identification of lipid species by mass spectrometry was based on their exact masses in the full scan HR-MS spectrum and MS/MS experiments. MS/MS fragmentation of choline-containing species in positive ion mode notably allowed the obtaining of structural information about these lipid species by identifying the polar-head group (characteristic and prominent fragment ion with *m/z* 184 [C_5_H_15_NO_4_P]^+^ corresponding to phosphocholine).

The levels of lipid species were calculated by normalizing the intensity of each individual species in the full scan spectrum to the intensity of corresponding internal standard, and multiplying by the spiked amount of the internal lipid standard. In addition, isotopic corrections were performed on the MS data in order to obtain more accurate measurements [17]. The total amount of a lipid class was calculated by summing the amounts of all individual species corresponding to this lipid class. All spectra were recorded with MassLynx software© (Version 4.1, Waters). Data processing for high-resolution full scan experiments was carried out by ALEX Software with the help of a Lipid Maps Lipidomics Gateway^®^ (https://www.lipidmaps.org/accessed on 2 January 2023). The data processing of MS/MS spectra was carried out using Biovia Draw 19.1© and MassLynx© software.

### 2.7. Statistical Analysis

Statistical analyses were performed using GraphPad Prism 5 software. The data are presented as mean ± standard deviation (SD). Significant differences between datasets were evaluated either by two-tailed *t*-tests (unpaired *t* test), by one-way ANOVA or Two-way ANOVA completed by adequate post-tests when appropriate. Variation is considered significant at a *p* value level less than or equal to 0.05. Correlative studies were carried out based on the non-parametric monotonic Spearman correlation. The correlation coefficient was calculated to determine associations between variables. Correlations were considered significant when the *p* value was less than or equal to 0.05.

## 3. Results

### 3.1. Identification of Choline-Containing Lysophospholipids and Phospholipids in Human Synovial Fluids (HSF)

Since LPC activates the ASIC3 channel [2,3], we first measured the amount of this choline-containing species in patient synovial fluids. In addition to LPC, we extended our research to PC and PC plasmalogen (PCp) in order to evaluate their potential roles in chronic joint pain. For this purpose, we optimized sample preparation conditions for a better recovery of these molecules in HSF.

**Figure 1:**
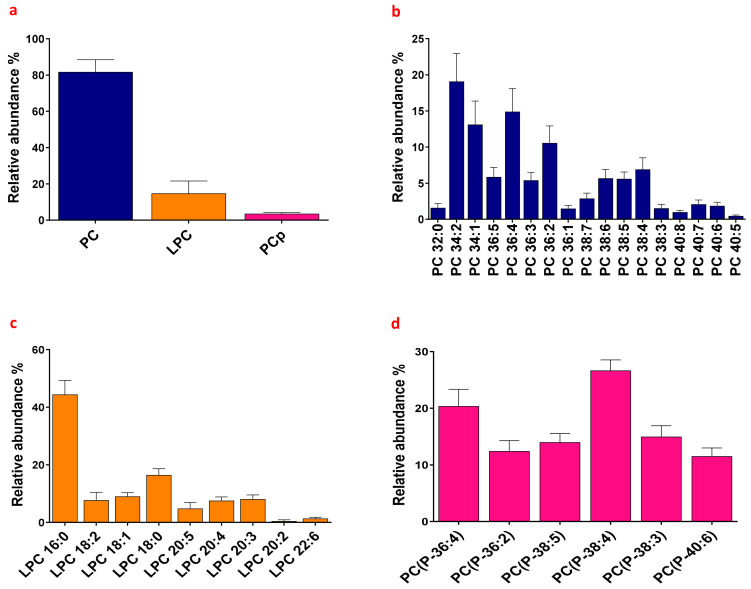
Relative abundances of analyzed lipids in human synovial fluids (HSF) of a cohort of patients suffering from joint pain associated with different rheumatic diseases. Analysis of lipid species in HSF samples was carried out by a direct infusion into mass spectrometry (MS) using an electrospray ionization source (ESI) in positive ion mode. (**a**) Relative abundances of lipid classes (PhosphatidylCholine PC, LysoPhosphatidylCholine LPC and plasmalogen PC PCp). (**b**) Relative abundances of PC species. (**c**) Relative abundances of LPC species showing a high level of LPC 16:0. (**d**) Relative abundances of the different PCp species.

Among identified lipids, PC was the most abundant lipid class, representing ~80% of total choline-containing lipids, whereas LPC accounted for ~15% and PCp for ~4% (Figure 1a). PCp displays a structure very similar to PC, which is only different due to a fatty alcohol with a vinyl-ether bond at the sn-1 position of the glycerol backbone [18]. In contrast to PC, PCp were found in very small amounts in HSF (~80% and ~4% of choline-containing lipids, respectively). Finally, we evaluated the presence of two additional choline-containing lipids in the synovial fluids, namely Platelet-activating factor (PAF) and its lyso-form, Lyso PAF, which have structures similar to PC and LPC, respectively. These molecules can be synthesized either de novo or by the remodeling pathways [19,20]. PAF was absent from HSF, while Lyso-PAF species were detected as traces in these samples (forming only ~0.5% of total choline-containing lipids). Due to their low abundances within HSF, PAF and Lyso-PAF were not considered further in the present study.

We next analyzed the relative abundances of the various molecular species within each lipid class. PC, PCp and LPC contain fatty acid chains that can vary in terms of both length (expressed as the number of carbon atoms) and number of double-bonds (or unsaturation). In Figure 1b–d, lipid subspecies are indicated as the type of lipid class (PC, LPC or PCp) followed by the total number of carbon atoms in the acyl chain (x) and the total number of double-bonds (y; e.g., PC x:y). As shown in Figure 1b, PC displayed a wide range of fatty acyl chain compositions: subspecies composed of saturated (y = 0), mono (y = 1) and poly (y ≥ 2)–unsaturated fatty acids containing 32 to 40 carbon atoms into the two acyl chains, with PC 34 and PC 36 being the most abundant. LPC species (which contain only one fatty acid) bearing the saturated chains palmitate (LPC 16:0) and stearate (LPC18:0) were the most abundant LPC, together forming more than 60% of total LPC (Figure 1c, data already published in [3]). Concerning PCp, they were exclusively composed of poly-unsaturated fatty acids with similar relative abundance (Figure 1d).

### 3.2. Identification of Potential Lipid Biomarkers in the Synovial Fluid of Patients Suffering from Chronic Joint Pain Compared to Control Synovial Fluids

**Figure 2:**
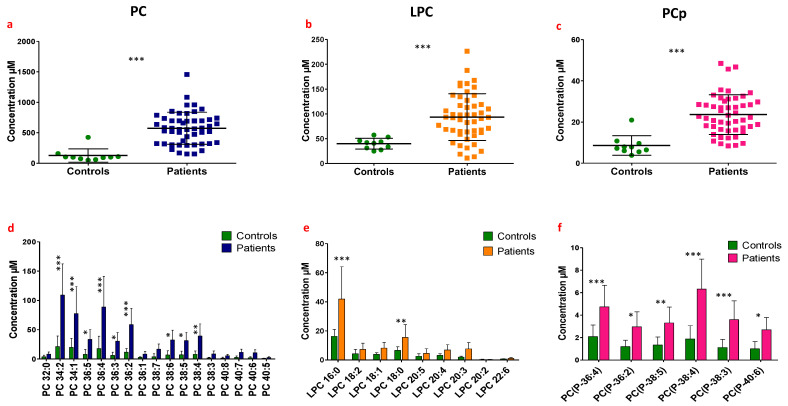
Comparison between lipid levels in HSF samples of the cohort of patients suffering from joint pain associated with different rheumatic diseases (Patients) and post-mortem controls (Controls). Lipids were extracted from HSF samples and the levels of PC, LPC and PCp species were measured based on ESI-MS analysis after the addition of lipid internal standards. The comparison between the total amount of each lipid class between the patients and the controls was based on an unpaired *t*-test, while the two-way ANOVA test was performed to compare multiple lipid species within each lipid class. (**a**,**d**) Histogram representing the comparison of the total PC concentration and individual PC species concentrations (µM) in HSF between patients and controls. (**b**,**e**) Histogram representing the comparison of total LPC concentration and individual LPC species concentrations between patients and controls. (**c**,**f**) Histogram representing the comparison of total PCp concentration and of individual PCp species concentrations between patients and controls. ns: not significant *p* > 0.05, *: 0.01 < *p* < 0.05, **: 0.001 < *p* < 0.01 and ***: *p* < 0.001.

Next, the levels of the various lipid species in synovial fluids of post-mortem controls were measured by mass spectrometry and compared to those of patients suffering from different rheumatic diseases. Global comparisons of total lipid concentrations and levels of individual species were first performed irrespective of the joint pathology. The total concentrations of choline-containing lipids (PC, LPC and PCp) were all significantly higher in patients compared to controls (Figure 2a, PC concentration of 573.2 ± 37.3 µM and 126.8 ± 34.4 µM in patients and controls, respectively, *p* < 0.0001; Figure 2b, LPC concentrations of 93.6 ± 6.66 µM and 40 ± 3.4 µM in patients and controls, respectively, *p* = 0.0007; [3] Figure 2c PCp concentrations of 23.6 ± 1.4 µM and 8.6 ± 1.5 µM in patients and controls, respectively, *p* < 0.0001).

Interestingly, the increase in total lipid concentrations differed between individual molecular species depending on the lipid class considered (PC, LPC and PCp). Individual PC and PCp molecular species exhibited an overall non-selective increase. The increase of total PC levels concerned nine individual species, bearing either saturated or unsaturated fatty acid chains and composed of 34, 36 and 38 carbon atoms on both chains (Figure 2d). The increase in total PCp levels was the result of an increase of all individual PCp species (Figure 2f). By contrast, the increase in total LPC was due to selective increases of the saturated species LPC16:0 and LPC18:0 (*p* < 0.001 and *p* < 0.01, respectively, [3]). The other LPC species did not display any significant variations compared to controls (Figure 2e).

### 3.3. Correlational Studies between Lipid Levels in Synovial Fluids and Patients’ Clinical Characteristics (Parameters)

**Figure 3:**
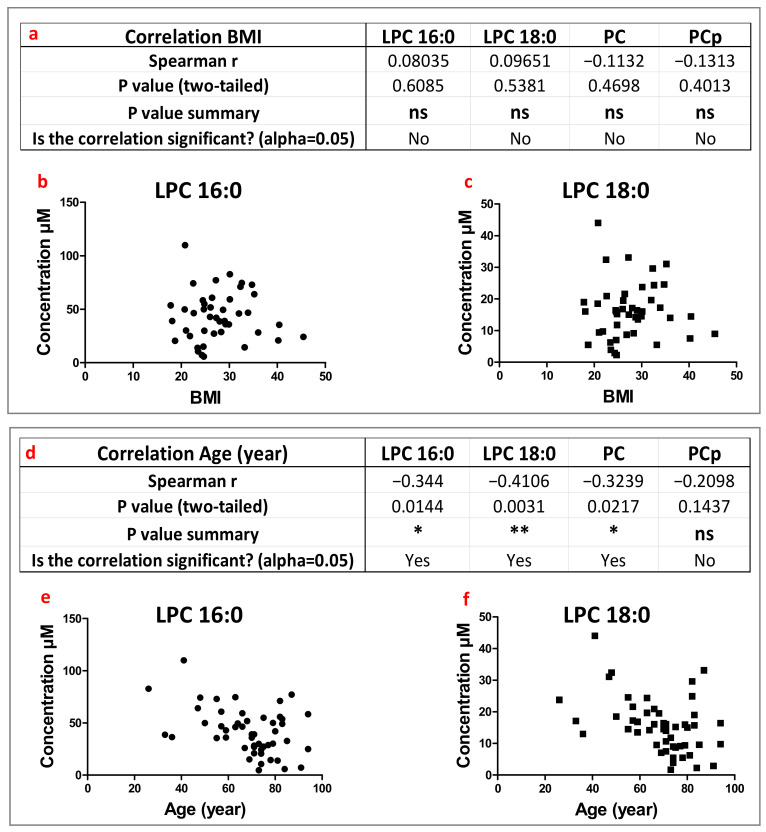
Correlation of lipid levels in HSF with the body mass index (BMI) and age of patients. (**a**), Table of Spearman correlation between lipid levels in HSF and BMI. (**b**,**c**) Correlation plots of BMI with LPC16:0 and LPC18:0, respectively. (**d**) Table of Spearman correlation between lipid levels in HSF and the age of patients regardless of joint pathologies. (**e**,**f**) Correlation plots of age with LPC16:0 and LPC18:0, respectively. ns: not significant *p* > 0.05, *: 0.01 < *p* < 0.05, and **: 0.001 < *p* < 0.01.

Potential correlations between lipid levels of patient synovial fluids and their clinical characteristics, including age, gender, and body mass index (BMI) were evaluated. Since PC and PCp showed non-selective increases among their various molecular subspecies in patients, correlative studies were performed on their total amounts. By contrast, since LPC16:0 and LPC18:0 were specifically involved in global LPC increases in patients, the correlational studies were focused on these selective species. No significant correlations have been observed between the amounts of these different lipid species with patients’ gender (Supplementary Figure S1a–d) or body mass index (BMI; Figure 3a–c). However, significant negative correlations between LPC16:0, LPC18:0, PC contents, and age were clearly observed (Figure 3d–f). To summarize, it appears that if the amounts of choline-containing lipids in synovial fluids seem independent of patients’ gender or BMI, concentrations of PC and its most represented lyso forms (LPC16:0 and LPC18:0) clearly decreased with patient age.

### 3.4. Correlation Studies between Lipid Levels in Patient Synovial Fluids, Type of Joint Pathologies and Inflammatory State

In order to assess whether selective lipid levels in patient synovial fluids could be potential markers of specific joint pathologies, we analyzed lipid changes according to joint diseases as compared to controls. The different joint diseases included inflammatory rheumatism (rheumatoid arthritis (RA), *n* = 6; psoriatic rheumatism (PA), *n* = 4; and spondyloarthritis (SPA), *n* = 5), microcrystalline arthropathies (chondrocalcinosis (CCA), *n* = 12 and Gout, *n* = 5) and osteoarthritis (OA, *n* = 18). Although we are aware that the number of patients for certain pathologies is a relatively limiting factor, the comparisons between lipid levels in synovial fluids still revealed interesting differences.

**Figure 4:**
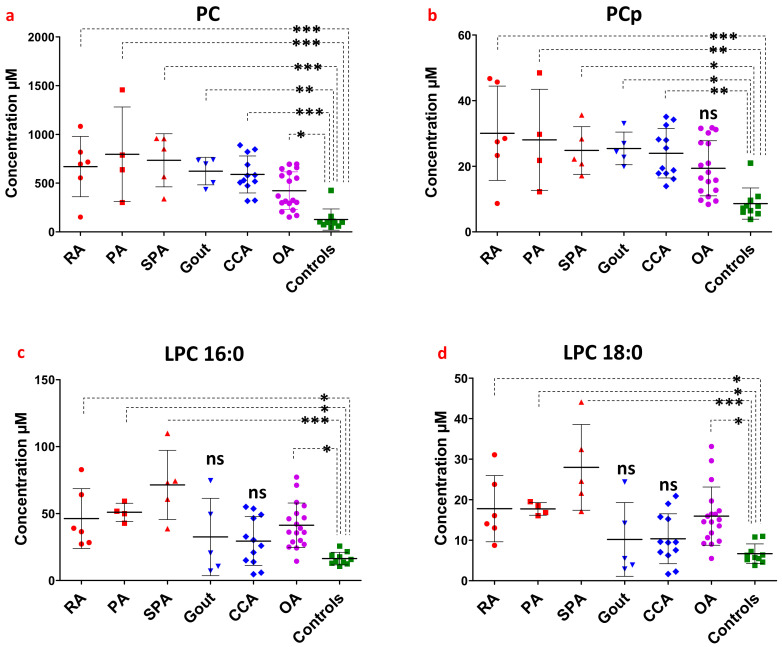
Evaluation of lipid concentrations in the synovial fluid of patients according to the rheumatic diseases associated with chronic joint pain. (**a**–**d**) Concentrations (in µM) of PC, PCp, LPC16:0, and LPC18:0, respectively, in the synovial fluid of patients depending on joint pathologies: (RA, *n* = 6), (Gout, *n* = 5), (CCA, *n* = 12), (PA, *n* = 4), (SPA, *n* = 5), (OA, *n* = 18) and controls *n* = 10. The comparison between lipid concentrations in the individual joint pathologies was based on the one-way analysis of variance completed with Bonferroni’s Multiple Comparisons Test. ns: not significant *p* > 0.05, *: 0.01 < *p* < 0.05, **: 0.001 < *p* < 0.01 and ***: *p* < 0.001.

When dividing patient subgroups according to their individual joint diseases, PC levels were significantly increased in all patient samples independently of the type of pathology (Figure 4a). A similar result was observed for PCp with increased concentrations in all patient subgroups, even if statistical significance was not reached for the OA group compared to controls (Figure 4b).

By contrast, LPC16:0 and LPC18:0 levels (Figure 4c,d) were only significantly increased in patients suffering from rheumatism with a strong inflammatory component (RA, PA and SPA) and OA, but not for individuals suffering from microcrystalline arthropathies (CCA and gout). Thus, high LPC contents, specifically the LPC16:0 and LPC18:0 species, in synovial fluids seem to be a potential hallmark of RA, PA, SPA and OA, whereas high PC and PCp levels were observed in patients suffering from all the different joint diseases included in our study.

**Figure 5:**
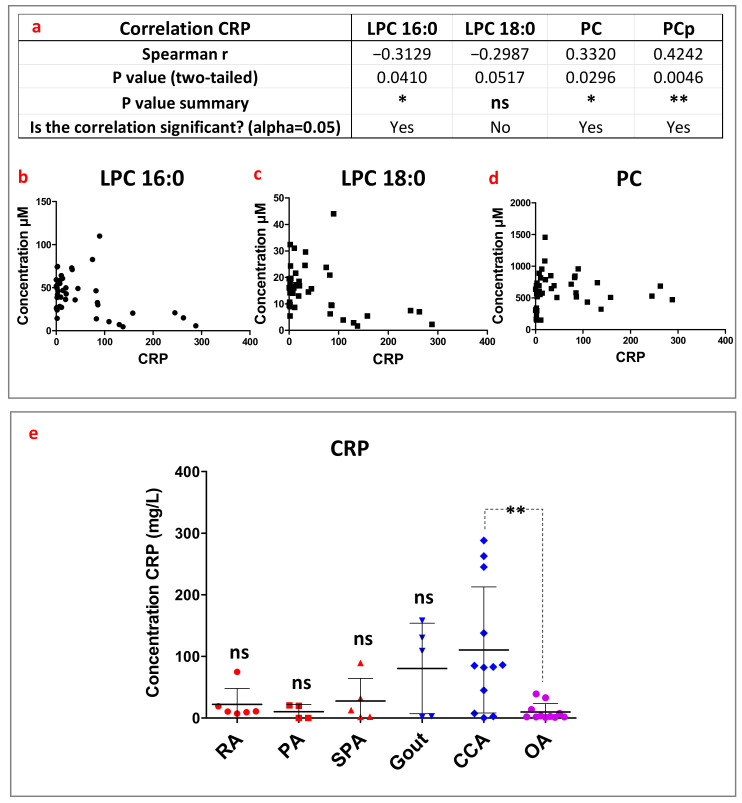
Lipid concentrations in the synovial fluid of patients according to the inflammatory state of the patients. Circulating C-Reactive Protein (CRP mg/l) was considered as an indicator of systemic inflammation. (**a**) Table of Spearman correlation results of circulating CRP with LPC16:0, LPC18:0, PC and PCp levels. (**b**–**d**), Correlation plots of CRP level with LPC16:0, LPC18:0, and PC levels, respectively. (**e**) Concentration (in mg/L) of circulating CRP according to the different joint diseases showing higher CRP levels in microcrystalline arthropathies patients. One-way analysis of variance completed with the Bonferroni’s Multiple Comparisons Test were performed to compare CRP concentrations in the individual joint pathologies; ns: not significant *p* > 0.05, *: 0.01 < *p* < 0.05, **: 0.001 < *p* < 0.01.

Since most of these joint diseases are known to be associated with systemic inflammation, we then evaluated whether a correlation could be observed between one of these choline-containing lipids and the concentrations of circulating C-reactive protein (CRP), used as a bona fide marker of systemic inflammation. This correlative study has been carried out on 43 patients whose CRP values were available. The levels of most of the synovial lipid species (e.g., LPC16:0, PC and PCp) correlated with the level of circulating CRP (Figure 5a), but, surprisingly, either positively or negatively depending on the lipid considered. LPC16:0 concentration tended to slightly decrease when the plasma CRP level increased with a negative correlation observed (r = −0.3129, *p* = 0.041, Figure 5a,b). LPC18:0 almost reached statistical significance (r = −0.2987, *p* = 0.0517, Figure 5a,c) with CRP. In contrast, total PC and PCp concentrations seem to increase when the CRP level increased, with positive correlations observed (r = 0.3320, *p* = 0.0296 for PC, and r = 0.4242, *p =* 0.0046 for PCp, Figure 5a,d).

We subsequently investigated whether certain joint pathologies were more specifically associated with higher levels of CRP. Patients with microcrystalline arthropathies appeared to have the highest CRP values (Figure 5e). The CCA patients were the only ones who showed significantly higher levels of CRP as compared to OA patients (Figure 5e). This result suggests an association of microcrystalline arthropathies, especially chondrocalcinosis, with chronic systemic inflammation. Moreover, high LPC16:0 and LPC18:0 contents, which were a potential hallmark of inflammatory rheumatism and OA (Figure 4c), seem to be disconnected from the inflammation status of patients, and even inversely correlated. It is worthy of note that the conclusion on the link between CRP and the different joint pathologies could be impacted by the treatment applied to the patients.

### 3.5. Possible Origins of High Levels of Selective Lipids in Patient Synovial Fluids

**Figure 6:**
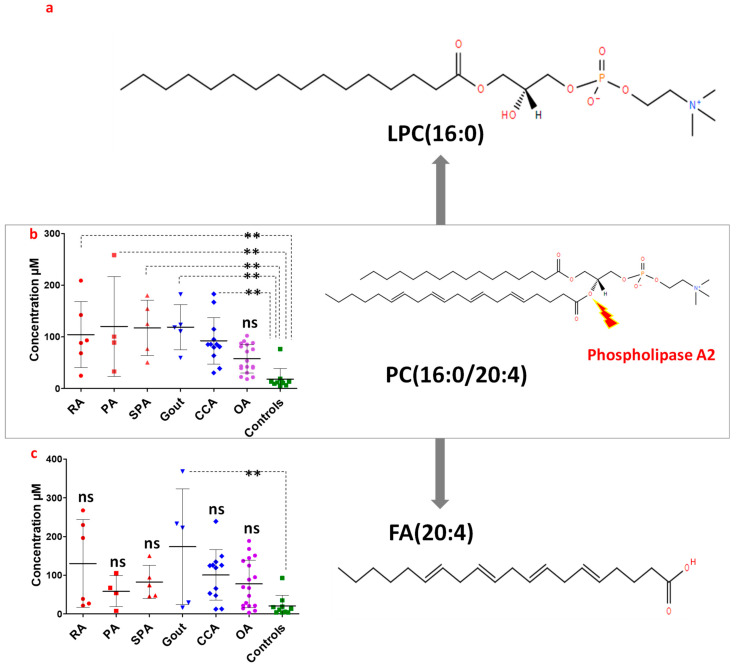
Evaluation of phospholipase A2 (PLA2) role in the variation of LPC16:0 levels in the synovial fluid of patients. (**a**) Represents the structure of LPC16:0. (**b**,**c**) Represents the concentration (in µM, left panel) and the structure (right panel) of PC(16:0/20:4) and FA20:4, respectively, in the different rheumatic diseases compared to controls. A one-way analysis of variance completed with the Bonferroni’s Multiple Comparisons Test were performed to compare lipid concentrations in the individual joint pathologies; ns: not significant *p* > 0.05, **: 0.001 < *p* < 0.01.

LPC16:0 and LPC18:0 are both derivatives of PC species through the action of the lysophosphatidylcholine acyltransferase (LPCAT), and the byproducts of selective PC species hydrolysis by phospholipase A2 ((PLA2); Figure 6 and Supplementary Figure S2) [6,21]. This hydrolysis of selective PC species bearing 16:0 or 18:0 chains at the sn-1 position and the fatty acid FA(20:4) at the sn-2 position (PC(16:0/20:4); Figure 6, and PC(18:0/20;4); Supplementary Figure S2), leads to the generation of LPC16:0 and LPC18:0, respectively, and of the free fatty acid 20:4 (FA20:4, also known as arachidonic acid, AA) [6]. Thus, phospholipase A2 (PLA2) is known to have a pro-inflammatory role since FA20:4 is a precursor of prostaglandins and leukotrienes [22,23].

We therefore postulated that high LPC16:0 and LPC18:0 levels observed in inflammatory rheumatism (RA, PA and SPA) and OA could be the result of the selective hydrolysis of local PC(16:0/20:4) and PC(18:0/20:4) pools, respectively, through the action of PLA2. In such a case, one would expect PC(16:0/20:4) and PC(18:0/20:4) levels (Figure 6b and Supplementary Figure S2b) to decrease at the expense of LPC16:0 and LPC18:0 (Figure 4c and Supplementary Figure S2a) and FA20:4. Therefore, FA20:4 content was also determined in the HSF (Figure 6c and Supplementary Figure S2c). Unexpectedly, high LPC16:0 and LPC18:0 levels in inflammatory rheumatism and OA patients were associated with elevated PC(16:0/20:4) and PC(18:0/20:4) concentrations as well as similar FA20:4 levels compared to controls (Figure 6 and Supplementary Figure S2). Moreover, for microcrystalline arthropathies (gout and CCA), both LPC16:0 (Figure 4c) and FA20:4 (Figure 6c) levels were clearly not matching with PC(16:0/20:4) levels (Figure 6b), arguing against a PLA2 origin of LPC16:0 and FA20:4. Similarly, PLA2 seems unrelated to the LPC18:0 increase observed in gout and CCA patients (Supplementary Figure S2). Taken together, these data suggest that increased LPC16:0 and LPC18:0 levels in inflammatory rheumatism (RA, PA and SPA) and OA are unlikely to result from the selective hydrolysis of PC(16:0/20:4) and PC(18:0/20:4) local pools by PLA2.

**Figure 7:**
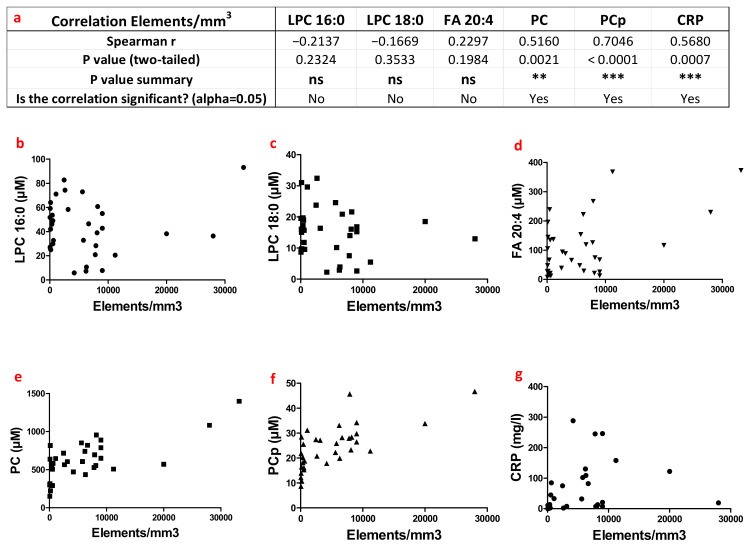
Assessment of potential associations between the number of cellular elements in human synovial fluids and the variation of lipid levels. (**a**) Table of Spearman correlation between the number of cellular elements per mm^3^ of synovial fluids and lipid concentrations (in µM) in the synovial fluids. (**b**–**g**) Correlation plots of the number of cellular elements with LPC16:0, LPC18:0, FA20:4, PC, PCp and C-Reactive Protein levels, respectively. ns: not significant *p* > 0.05, **: 0.001 < *p* < 0.01 and ***: *p* < 0.001.

To further investigate the potential origins of these increased lipid levels in patients’ HSF, we performed a wider correlative study between lipid levels and other factors in the synovial fluids and plasma of patients (see Section 2). Among the variables studied, the number of cellular elements in patient synovial fluids appeared to be highly correlated with the levels of some choline-containing lipids. Cellular elements in patients HSF mainly correspond to inflammatory cells that are recruited to the synovial fluid and therefore reflect the level of local inflammation. Under non-pathological conditions, synovial fluids exhibit < 200 cells/mm^3^, while inflammatory synovial fluids can contain up to 1000–2000 cells per mm^3^ [24]. Even if there was no significant relationship between LPC16:0, LPC18:0 or FA20:4 levels and the number of cellular elements per mm^3^ (Figure 7a–d), strong positive correlations were observed between this parameter and PC and PCp levels (r = 0.516, *p* = 0.0021, *n* = 33 for PC, r = 0.705, *p <* 0.0001, *n* = 33 for PCp, Figure 7a,e,f). Moreover, plasma CRP concentrations, which have already been positively correlated with PC and PCp levels in synovial fluids, also displayed a strong positive correlation with the number of cellular elements per mm3 in HSF (r = −0.568, *p =* 0.0007, *n* = 32, Figure 7a,g).

Besides the number of cellular elements in synovial fluids, several blood parameters defining patients’ metabolic status appeared to correlate with selective lipid levels in their synovial fluids. Correlative studies using the Spearman’s monotonic function were performed with several circulating lipids/lipoproteins (cholesterol, high-density lipoprotein (HDL), low-density lipoprotein (LDL) and triglyceride (TG)) and blood glucose, as bona fide indicators of patient metabolic status (Table 1). Interestingly, total LPC, LPC16:0 and LPC18:0 levels strongly correlated with circulating concentrations of Cholesterol, HDL and LDL, but not with glucose (Table 1). An intermediate result was observed with TG, with no correlation for total LPC and LPC18:0 but a moderate positive correlation with LPC16:0. Interestingly, PC and PCp levels did not correlate with any of these metabolic parameters (Table 1).

Altogether, our data revealed that synovial PC and PCp levels positively correlated with the inflammatory status of the patient, evaluated either by circulating CRP levels (Figure 5) or by the number of synovial cellular elements (Figure 7). In contrast, PC and PCp contents appeared to be relatively independent of patients’ metabolic status (Table 1). Interestingly, the opposite was observed for the levels of synovial LPC species, which appeared to strictly correlate with circulating metabolic markers and more specifically with the lipid status of the patient (Table 1), while being uncorrelated with inflammatory processes (Figure 5 and Figure 7).

## 4. Discussion

LPC, and especially LPC16:0, has been shown to directly activate the ASIC3 channel to generate chronic joint pain [2,3]. In our recent published work, we observed higher levels of total LPC in the synovial fluids of two independent cohorts of patients, including a first cohort of OA patients and a second of patients with different joint pathologies, than in post-mortem controls [3]. This increase in LPC contents was related to a specific increase of the LPC16:0 species, which correlated with OA patients’ pain outcomes [3]. Here, we used synovial samples from the second cohort of patients suffering from different painful joint diseases [3] to identify and measure the levels of choline-head lipids compared to controls, using HR-MS analysis methods.

As reported in our previous study [3], we observed higher levels of total LPC in patient synovial fluids compared to controls, regardless of the joint pathologies they suffered from. This increase in total LPC was very selective to the saturated species LPC 16:0, and, to a lower extent, LPC18:0. Interestingly, we identified higher levels of total PC and PCp in the synovial fluids of patients compared to controls in the present study. In contrast to LPC, the increase of PC and PCp levels was associated with a global non-selective augmentation of most individual PC and PCp species, containing either saturated or unsaturated fatty acyl chains. These latest results are in agreement with previous studies which also reported higher levels of most phospholipids in the synovial fluids of RA patients [12,14], and early and late OA patients [13,15], compared to the synovial fluids of healthy controls. However, lipid profiling in the synovial fluids of patients suffering from other types of joint pathologies was still missing.

In this study, we observed differences between lipid profiles depending on the type of joint disease considered. If PC and PCp levels appeared to be non-selectively increased in all types of patients compared to controls, we found a specific increase in LPC16:0 and LPC18:0 levels in patients suffering from OA and inflammatory rheumatism (namely RA, PA and SPA). In contrast, patients with microcrystalline arthropathies (gout and CCA) exhibited similar LPC16:0 and LPC18:0 levels as controls.

Since most of these pathologies are associated with local and/or systemic inflammation, we investigated whether specific lipid signatures could correlate with the concentrations of cellular elements in synovial fluids and/or with circulating CRP levels used as markers of local and systemic inflammation, respectively. It has already been reported that the levels of circulating CRP correlate with CRP concentrations in the synovial fluid following periprosthetic joint infection [25], and in several joint diseases such as RA, OA and PA [26]. Generally, inflammatory joint pathologies are associated with elevated levels of circulating CRP, as already shown in RA patients [27]. However, the relationship between the concentration of circulating CRP and lipid levels in synovial fluids needed to be addressed. In this study, we observed positive correlations between the levels of PC and PCp in HSF with both circulating CRP concentrations and the number of HSF cellular elements. Therefore, it seems that the concentrations of these choline-containing lipids are intimately linked to the inflammatory status of patients. Since PC and PCp are major components of cellular membranes, their increase in patients’ synovial fluids may reflect the recruitment of cells from the immune system at the joint level and, ultimately, the intensity of the inflammatory processes.

Interestingly, a very different situation was observed for LPC species. The levels of total LPC, especially of the selective saturated species LPC16:0 and LPC18:0, did not positively correlate with the concentration of cellular elements in synovial fluids, nor with circulating CRP levels. LPC16:0 was even found to be negatively correlated with CRP levels. These data strongly suggest that LPC levels in the synovial fluids are largely independent of the inflammatory status of patients. To the best of our knowledge, such an observation has not been reported previously in painful joint diseases. At first glance, this finding seems to contradict the pro-inflammatory effects of LPC [28,29,30,31,32], especially the saturated species, observed in several studies [28,33]. However, a similar observation to ours has been made, where decreased levels of circulating LPC have been observed in sepsis patients compared to controls [34]. In good agreement with this latest observation, it has been suggested that LPC may exert an immuno-suppressive function through its binding to Immunoglobulin G2A [34,35]. Other studies also demonstrated that the administration of selective LPC species can have an anti-inflammatory effect in chronic inflammatory diseases [34,36,37,38], including RA [39]. In brief, if the role of LPC species in inflammation still remains controversial, it appears here that LPC levels in HSF did not parallel the inflammatory processes, clearly differentiating it from other choline-containing lipids, such as PC and PCp.

In order to identify the origin of elevated LPC16:0 and LPC18:0 concentrations in patient synovial fluids, we evaluated the potential relationship between LPC16:0 and LPC18:0 and a dysregulation of PLA2 activity. Indeed, PLA2 catalyzes the hydrolysis of PC species to concomitantly generate a LysoPC and a free fatty acid. Under inflammatory conditions, PLA2 preferentially cleaves PC(16:0/20:4) and PC(18:0/20:4) to generate LPC16:0 and LPC18:0, respectively, and AA (FA20:4), a precursor of prostaglandin and leukotrienes, both acting as pro-inflammatory mediators [22,23]. If such a situation occurs in patients (i.e., induction of PLA2 activity), one would expect LPC16:0, LPC18:0, and FA20:4 levels to increase at the expense of PC(16:0/20:4) and PC(18:0/20:4). However, such a scenario was never observed, regardless of the joint diseases considered, suggesting that increased LPC16:0 and LPC18:0 contents are unlikely to primarily result from the hydrolysis of local PC(16:0/20:4) and PC(18:0/20:4) pools by PLA2.

We also extended our correlation studies to other clinical patient traits including gender, age, BMI, and several metabolic parameters. Levels of choline-containing lipids appeared to be largely disconnected from gender and BMI. However, a negative correlation was observed between the amounts of LPC and PC species and patient age. If no clear explanation has emerged so far, knowing the impact of LPC16:0 on the activation of ASIC3 channels, one may expect that older individuals would be less sensitive to chronic joint pain. However, even if old patients’ groups reported better mood and quality of life than young and middle-aged groups in previous studies, pain intensity did not vary meaningfully as a function of age [40]. Therefore, additional studies will be required to address the possible connections between pain intensity, age and LPC levels in the synovial fluids of patients suffering from chronic joint pain.

Another very intriguing observation was that total LPC, LPC16:0 and LPC18:0 concentrations positively correlated with circulating lipid/lipoprotein markers (cholesterol, HDL and LDL), whereas PC and PCp levels in HSF were largely disconnected from the metabolic status of patients. Accordingly, Oliviero et al. have reported positive correlations between the levels of lipoprotein in the serum and neutral lipids in the synovial fluid of patients suffering from various joint diseases [41]. Joint pain is known to be worse in the case of metabolic syndrome [42,43]. Potential connections between the level of pain-inducing lipids (i.e., LPC) in chronic joint pain and the global dysregulation of lipid metabolism related to metabolic syndrome appears to be a very attractive hypothesis to pursue.

Finally, we further reported the absence of high LPC levels in the synovial fluids of patients with microcrystalline arthropathies [3], in contrast to the other joint diseases. Interestingly, we observed higher levels of AA (FA20:4) in patients suffering from gout, and to a lower extent (and at the limit of significance), in other joint diseases compared to controls. It has to be noted that AA is, like LPC16:0, a fairly good activator/potentiator of the ASIC3 channel [2]. Therefore, studying the synergistic effects of these two-lipid species on ASIC3 activity and their relations/contributions to pain intensity will definitely bring new avenues in the understanding and treatment of chronic joint pain.

## Figures and Tables

**Table 1:** Results of the Spearman correlative study between plasmatic concentrations of (cholesterol, HDL, LDL, TG and glucose), and lipid levels in the synovial fluids of the same patients irrespective of the rheumatic diseases.

			Synovial Fluid Lipids				
			LPC 16:0	LPC 18:0	LPC	PC	PCp
Plasma lipids	Cholesterol g/L	Spearman r	0.7088	0.6588	0.6971	0.4853	0.02059
		*p* value	0.0021	0.0055	0.0027	0.0567	0.9397
		*p* value summary	**	**	**	ns	ns
	HDL g/L	Spearman r	0.6577	0.6220	0.6434	0.3628	0.1948
		*p* value	0.0077	0.0133	0.0097	0.1838	0.4866
		*p* value summary	**	*	**	ns	ns
	LDL g/L	Spearman r	0.5754	0.6196	0.5946	0.4268	0.09860
		*p* value	0.0197	0.0105	0.0151	0.0992	0.7164
		*p* value summary	*	*	*	ns	ns
	TG	Spearman r	0.5063	0.3297	0.4474	0.2693	−0.2870
		*p* value	0.0454	0.2124	0.0823	0.3131	0.2812
		*p* value summary	*	ns	ns	ns	ns
	Blood glucose plasma	Spearman r	−0.4476	−0.5245	−0.5035	−0.2727	−0.5105
		*p* value	0.1446	0.0800	0.0952	0.3911	0.0899
		*p* value summary	ns	ns	ns	ns	ns

ns: not significant *p* > 0.05, *: 0.01 < *p* < 0.05, **: 0.001 < *p* < 0.01.

## Data Availability

The authors declare that all supporting data are available within the article.

## Supplementary Materials

The following supporting information can be downloaded at: https://www.mdpi.com/article/10.3390/biom13020342/s1, Figure S1: Correlation of lipid levels in the synovial fluid of patients with gender. Figure S2: Evaluation of phospholipase A2 (PLA2) role in the variation of LPC18:0 level in the synovial fluid of patients. Table S1: Age (year) and BMI distribution in patients according to the different joint pathologies associated with chronic joint pain, Table S2: Optimized parameters of the Electrospray Ionization Source ESI, coupled to the SYN-APTTM G2 MS, in positive and negative ion modes allowing the detection of the different lipid classes and molecular species, Table S3. A detailed list of lipids identified in human synovial fluids.
