# Supplementary material for: Identification of Lipid Biomarkers for Chronic Joint Pain Associated with Different Joint Diseases
Source: Biomolecules. 2023 Feb 9;13(2):342. doi: 10.3390/biom13020342 (PMC9953120; doi:10.3390/biom13020342)
Supplement: Supplementary file 1 [file biomolecules-13-00342-s001.zip › biomolecules-2170714-supplementary-final online.pdf]

biomolecules

Identification of lipid biomarkers for chronic joint pain associated with different joint diseases

Spiro Khoury *et al,*. 2023

Supplementary Materials

Figure S1

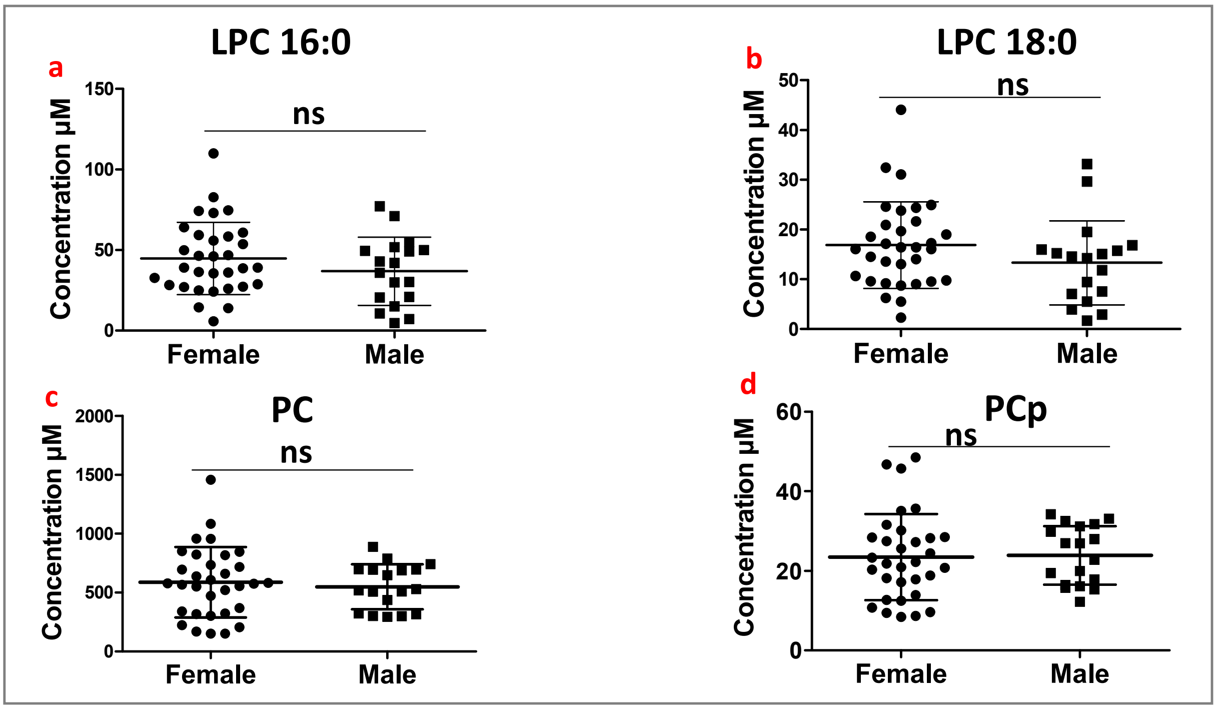

Figure S1. Correlation of lipid levels in the synovial fluid of patients with gender. **a, b, c** and **d** Comparisons of lipid concentrations (LPC16:0, LPC18:0, PC and PCp, respectively) between female and male showing no significant variation in lipid levels.

Figure S2

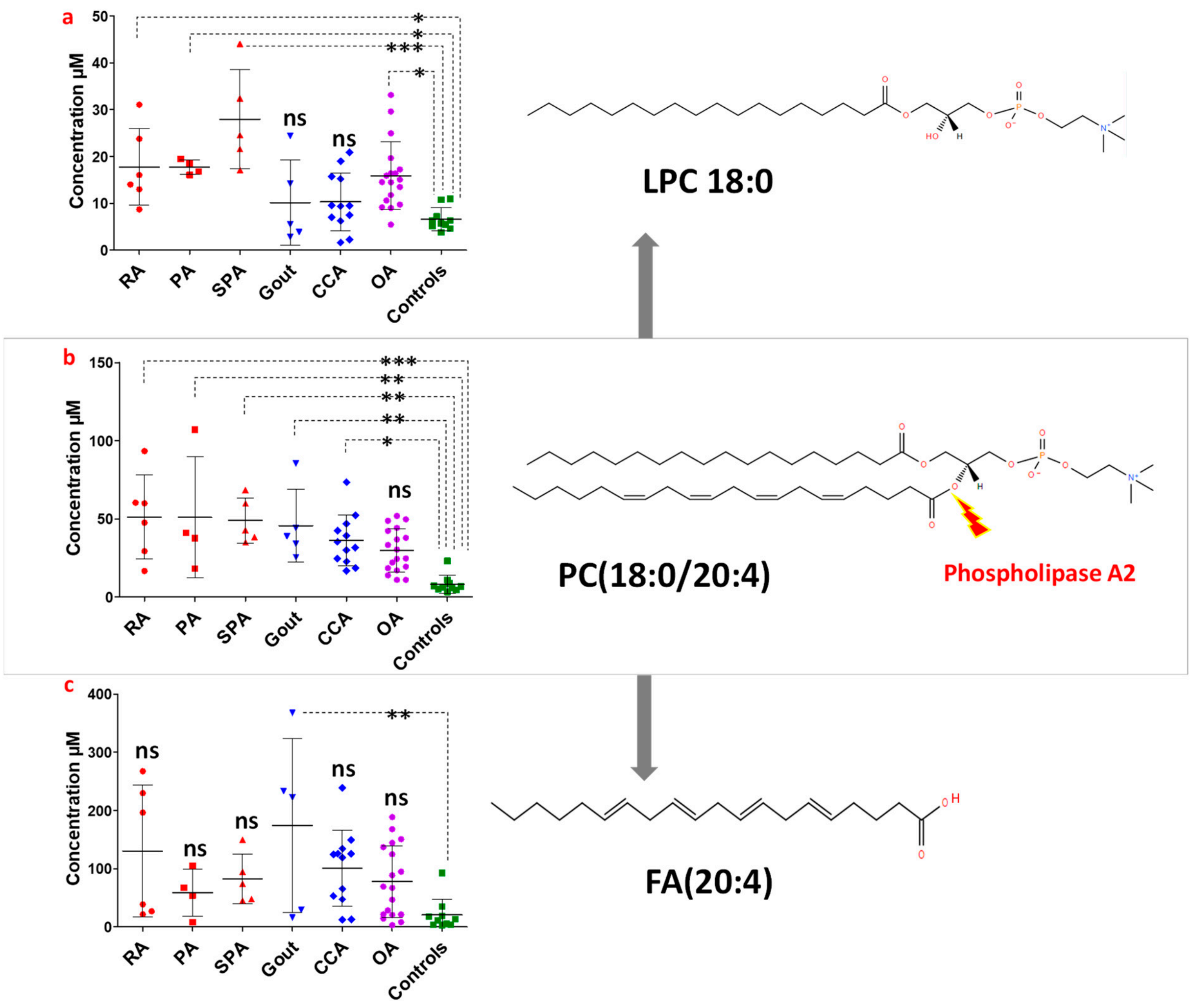

Figure S2. Evaluation of phospholipase A2 (PLA2) role in the variation of LPC18:0 level in the synovial fluid of patients. **a**, **b** and **c** Represent the concentration (in µM, left panel) and the structure (right panel) of LPC18:0, PC(18:0/20:4), FA20:4 , respectively, in the different rheumatic diseases compared to controls. The comparison between lipid concentrations in the individual joint pathologies was based on the one-way analysis of variance completed with the Bonferroni's Multiple Comparison Test ns: not significant $p > 0.05$, *: $0.01 < p < 0.05$, **: $0.001 < p < 0.01$ and ***: $p < 0.001$.

Table S1

Table S1. Age (year) and BMI distribution in patients according to the different joint pathologies associated with chronic joint pain. Values are expressed as average ± SD (rheumatoid arthritis RA; $n = 6$, psoriatic arthritis PA; $n = 4$, spondyloarthritis SPA; $n = 5$, chondrocalcinosis CCA; $n = 12$, Gout; $n = 5$, and osteoarthritis OA; $n = 18$).

Joint Pathology	Age (Average ± SD)	BMI (Average ± SD)
RA	54.3 ± 19	29.2 ± 6.5
PA	60.8 ± 7	25.7 ± 3.3
SPA	46.8 ± 8.9	26.5 ± 4.9
CCA	76.3 ± 6.7	24.9 ± 6.2
Gout	73.2 ± 10.1	25.6 ± 4.7
OA	74.7 ± 11.1	30.2 ± 5.9

Table S2

Table S2. Optimized parameters of the Electrospray Ionization Source ESI, coupled to the SYNAPT™ G2 MS, in positive and negative ion modes allowing the detection of the different lipid classes and molecular species.

Parameters	Positive ionization	Negative ionization
**Capillary voltage (kV)**	5	2.5
**Sampling Cone (V)**	50	70
**Extraction Cone (V)**	1.5	4
**Source Temperature (°C)**	80	120
**Desolvation Temperature (°C)**	75	150
**Con Gas (L/h)**	**25**	**100**
**Desolvation Gas (L/h)**	**50**	**500**

Table S3

Table S3. A detailed list of lipids identified in human synovial fluids.

Lipid species	Sum formula	Target m/z	Average measured m/z	Mass shift
LPC 16:0	C24H51NO7P	496.3398	496.3369	0.0028
LPC 18:2	C26H51NO7P	520.3398	520.3349	0.0048
LPC 18:1	C26H53NO7P	522.3554	522.3536	0.0018
LPC 18:0	C26H55NO7P	524.3711	524.3680	0.0031
LPC 20:5	C28H49NO7P	542.3241	542.3200	0.0042
LPC 20:4	C28H51NO7P	544.3398	544.3352	0.0045
LPC 20:3	C28H53NO7P	546.3554	546.3512	0.0042
LPC 20:2	C28H55NO7P	548.3711	548.3566	0.0144
LPC 22:6	C30H51NO7P	568.3398	568.3354	0.0043
PC 32:0	C40H81NO8P	734.5694	734.5655	0.0039
PC 34:2	C42H81NO8P	758.5694	758.5650	0.0045
PC 34:1	C42H83NO8P	760.5851	760.5801	0.0050
PC 36:5	C44H79NO8P	780.5538	780.5476	0.0062
PC 36:4	C44H81NO8P	782.5694	782.5634	0.0061
PC 36:3	C44H83NO8P	784.5851	784.5772	0.0079
PC 36:2	C44H85NO8P	786.6007	786.5960	0.0047
PC 36:1	C44H87NO8P	788.6164	788.6083	0.0081
PC 38:7	C46H79NO8P	804.5538	804.5457	0.0081
PC 38:6	C46H81NO8P	806.5694	806.5637	0.0057
PC 38:5	C46H83NO8P	808.5851	808.5786	0.0065
PC 38:4	C46H85NO8P	810.6007	810.5941	0.0067
PC 38:3	C46H87NO8P	812.6164	812.6094	0.0069
PC 40:8	C48H81NO8P	830.5694	830.5613	0.0082
PC 40:7	C48H83NO8P	832.5851	832.5774	0.0077
PC 40:6	C48H85NO8P	834.6007	834.5942	0.0065
PC 40:5	C48H87NO8P	836.6164	836.6075	0.0089
PC(P-36:4)	C44H81NO7P	766.5745	766.5694	0.0051
PC(P-36:2)	C44H85NO7P	770.6058	770.5959	0.0099
PC(P-38:5)	C46H83NO7P	792.5902	792.5810	0.0092

PC(P-38:4)	C46H85NO7P	794.6058	794.5983	0.0076
PC(P-38:3)	C46H87NO7P	796.6215	796.6086	0.0129
PC(P-40:6)	C48H85NO7P	818.6058	818.5933	0.0125
